# Supplementary material for: Regional Characterization Study of Fatty Acids and Tocopherol in Organic Milk as a Tool for Potential Geographical Identification
Source: Foods. 2020 Nov 26;9(12):1743. doi: 10.3390/foods9121743 (PMC7759942; doi:10.3390/foods9121743)
Supplement: Supplementary file 1 [file foods-09-01743-s001.zip › foods-982744-supplementary-1.pdf]

Eigenvalues table

Function	Eigenvalue	% of variance	Cumulative %	Canonical correlation
1	99.215[a]	73.5	73.5	0.995
2	22.324[a]	16.5	90	0.978
3	13.491[a]	10	100	0.965

Wilks' Lamda

Test of functions	Wilks' Lamda	Chi-square	df	Sig.
1 to 3	0.0000	928.3030	48.0000	0.0000
2 to 3	0.0030	518.2520	30.0000	0.0000
3	0.0690	237.9470	14.0000	0.0000

Structure matrix table

	Function		
	1	2	3
**α-tocopherol[a]**	**0.232***	-0.005	-0.203
C13:0	**0.133***	-0.084	-0.079
**C20:3 n-3 *all cis* [a]**	**0.079***	0.032	-0.039
C24:0	-0.031	**0.449***	0.297
C18:3 n-6 *all cis*	0.085	**0.166***	0.135
**C15:1 n-5 *cis* [a]**	-0.039	**-0.128**	-0.03
**C22:2 n-6 *all cis* [a]**	0.04	**-0.101**	-0.046
C18:2 n-6 *all cis*	0.035	**0.083***	-0.068
**C20:3 n-6 *all cis* [a]**	0.036	**0.076***	0.038
**C14:1 n-5 *cis* [a]**	-0.125	0.104	**-0.446**
C18:0	0.088	-0.016	**0.368***
**C16:1 n-7 *cis* [a]**	-0.082	0.038	**-0.361**
C17:0	0.323	0.032	**0.360***
C16:0	-0.103	-0.102	**0.356***
**C18:3 n-3 *all cis* [a]**	0.03	0.183	**-0.314**
**C18:1 n-9 *cis+trans* [a]**	-0.019	0.007	**-0.29**
**C22:1 n-9 *cis* [a]**	0.075	0.107	**-0.26**
**C20:2 n-6 *all cis* [a]**	0.015	0.093	**0.174***
**C22:6 n-3 *all cis* [a]**	0.099	0.12	**-0.162**
C12:0	-0.046	-0.017	**-0.15**
**C22:0[a]**	0.095	0.104	**-0.146**
C17:1 n-7 *cis* [a]	-0.009	-0.062	**-0.141**
C20:5 n-3 *all cis*	0.028	0.087	**-0.136**
C20:0	0.114	0.069	**-0.126**
C20:1 n-9 *cis*	0.055	0.09	**-0.119**
C10:0	0.011	0.002	**-0.117**
C14:0	-0.006	0.019	**-0.092**
C8:0	-0.009	0.012	**-0.086**
**C20:4 n-6 *all cis* [a]**	-0.049	-0.01	**0.052***

**C23:0**[a]	-0.041	0.007	**-0.045**
C11:0	0.029	-0.009	**-0.044**
**C6:0**[a]	-0.014	0.025	**0.042**[*]
C6:0	-0.004	0.01	**-0.018**

*Pooled within-groups correlations between discriminating variables and standardized canonical discriminant functions

a Variables ordered by absolute size of correlation within function.

Functions at group centroids table

Region	Function		
	1	2	3
Hoengseong	4.535	-6.34	-3.43
Boryeong	4.202	6.741	-2.999
Gochang	7.973	-0.254	5.493
Jeju	-16.709	-0.147	0.936

Unstandardized canonical discriminant functions evaluated at group means.

Box's M test results table

Box's M		2390.388
F	Approx.	4.064
	df1	408
	df2	19463.815
	sig,	**0.0000**

Tests null hypothesis of equal population covariance matrices.

Canonical Discriminant Function Coefficients table

	Canonical discriminant function coefficients		
	1	2	3
C6:0	2.141	-1.329	0.502
C8:0	-4.989	12.761	-1.432
C10:0	3.583	-4.697	0.759
C11:0	-6.135	2.802	3.273
C12:0	-1.837	-1.875	0.751
C13:0	5.064	-0.426	21.433
C14:0	-0.511	1.104	-0.816
C16:0	-0.04	-0.092	0.234
C17:0	6.303	0.162	-1.777
C18:0	-0.183	-0.207	0.236
C18:2 n-6 *all cis*	-0.419	0.222	-0.229
C18:3 n-6 *all cis*	-6.725	7.464	7.053
C20:0	5.12	3.864	-1.482
C20:1 n-9 *cis*	2.333	-0.001	0.05
C20:5 n-3 *all cis*	8.166	-1.416	4.406
C24:0	-1.843	5.53	3.647

(상수)	14.583	-39.447	-56.085

Unstandardized coeffi cients.

Standardized Canonical Discriminant Function

	Function		
	1	2	3
**C6:0**	**3.093**	-1.92	0.726
**C8:0**	**-4.243**	**10.852**	-1.218
**C10:0**	**7.187**	**-9.421**	1.521
C11:0	-0.796	0.364	0.425
**C12:0**	**-4.121**	**-4.206**	1.685
C13:0	0.348	-0.029	1.475
**C14:0**	**-2.068**	**4.469**	-3.306
C16:0	-0.203	-0.465	1.177
C17:0	1.993	0.051	-0.562
C18:0	-1.365	-1.541	1.756
C18:2 n-6 cis	-0.896	0.476	-0.49
C18:3 n-6 *all cis*	-0.371	0.412	0.389
C20:0	0.928	0.7	-0.269
C20:1	1.007	-0.001	0.022
C20:5 n-3 *all cis*	0.605	-0.105	0.326
C24:0	-0.397	1.191	0.786
