# Supplementary material for: Regional Characterization Study of Fatty Acids and Tocopherol in Organic Milk as a Tool for Potential Geographical Identification
Source: Foods. 2020 Nov 26;9(12):1743. doi: 10.3390/foods9121743 (PMC7759942; doi:10.3390/foods9121743)
Supplement: Supplementary file 1 [file foods-09-01743-s001.zip › foods-982744-supplementary-2.pdf]

[Supplementary Materials] including 1 Table and 1 Figure

Regional Characterization Study of Fatty Acids and Tocopherol in Organic Milk as a Tool for Potential Geographical Identification

Ill-Min Chung, Yun-Ju Kim, Hee-Sung Moon, Chang Kwon, Hee-Youn Chi, and Seung-Hyun Kim*

Department of Crop Science, College of Sanghuh Life Science, Konkuk University, Seoul 05029, Republic of Korea

Ill-Min CHUNG: imcim@konkuk.ac.kr,

Yun-Ju KIM: bluebeing123@konkuk.ac.kr,

Hee-Sung MOON: hsmoon7575@konkuk.ac.kr,

Chang KWON: chang794@konkuk.ac.kr,

Hee-Youn CHI: **chi1143@konkujk.ac.kr,**

Seung-Hyun KIM: kshkim@konkuk.ac.kr

* To whom correspondence should be addressed:

Tel: +82-02-2049-6163; Fax: +82-02-455-1044; E-mail: kshkim@konkuk.ac.kr

Running title: Geographic identification of organic milk in Korea

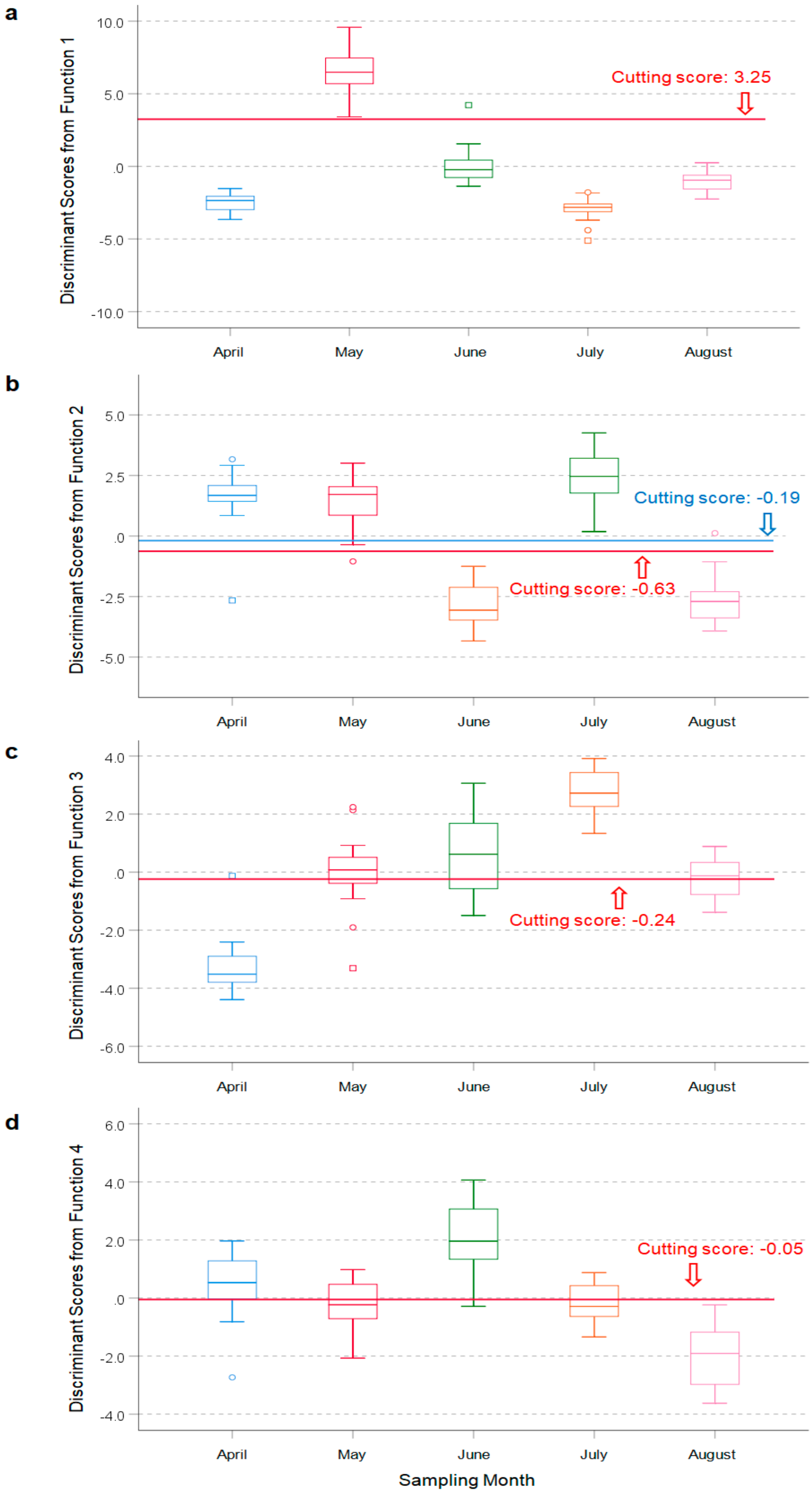

Figure S1. Box-and-whisker plots for discriminant scores from four canonical discriminant functions of the discriminative model (see Fig. 2b) developed for OM production month authenticity.

Table S1. Classification and cross-validated results[a,b] of OM production month by SDA.

			Predicted Group Membership					Total
			April	May	June	July	August	
Original	Count	April	19	0	0	0	1	20
		May	0	20	0	0	0	20
		June	0	0	19	0	1	20
		July	0	0	0	20	0	20
		August	0	0	0	0	20	20
	%	April	95	0	0	0	5	100
		May	0	100	0	0	0	100
		June	0	0	95	0	5	100
		July	0	0	0	100	0	100
		August	0	0	0	0	100	100
Cross-validated[c]	Count	April	19	0	0	0	1	20
		May	0	20	0	0	0	20
		June	0	1	18	0	1	20
		July	0	0	0	20	0	20
		August	1	0	1	0	18	20
	%	April	95	0	0	0	5	100
		May	0	100	0	0	0	100
		June	0	5	90	0	5	100
		July	0	0	0	100	0	100
		August	5	0	5	0	90	100

a. 98.0% of original grouped cases correctly classified.
b. 95.0% of cross-validated grouped cases correctly classified.
c. Cross validation is done only for those cases in the analysis. In cross validation, each case is classified by the functions derived from all cases other than that case.
